# Supplementary material for: Are carbon emissions trading and green financial instruments synergistic? -Comprehensive quantitative research based on content analysis
Source: PLoS One. 2024 Mar 7;19(3):e0298601. doi: 10.1371/journal.pone.0298601 (PMC10919601; doi:10.1371/journal.pone.0298601)
Supplement: S1 Data — (ZIP) [file pone.0298601.s001.zip › Data/地级市Z_F工作报告.zip!/╝¬┴╓/╦╔╘¡╩╨/└┤╫╘╣1⁄2╓┌║┼í╛┬φ┐╦╩2╛▌═°í┐_2015.docx]

各位代表：

　　现在，我代表市人民政府，向大会作工作报告，请予审议，并请各位政协委员提出意见。

　　一、2014年工作回顾

　　过去一年，是我市实施“十二五”规划关键一年，也是我市发展进程中很不平凡的一年。面对宏观经济环境趋紧、油气量价齐跌等多重挑战与考验，在省委、省政府和市委的坚强领导下，全市各族人民紧紧围绕“转型发展、富民强市，建设幸福松原”，砥砺攻坚，开拓奋进，科学调整并完成了计划指标。预计全市地区生产总值实现1740亿元，同比（下同）增长6.8%（不变价）；地方级财政收入完成62.5亿元，增长0.8%；全社会固定资产投资完成1168亿元，增长15%；社会消费品零售总额实现555亿元，增长7.8%；城镇和农村常住居民人均可支配收入分别达到26850元和11000元，分别增长3.5%和9.9%。这一年，我们努力加快经济转型，着力改善城乡面貌，推进全面深化改革，持续增进民生福祉，全市各项事业协调发展，社会保持和谐稳定，朝着全面建成小康社会又迈进了坚实一步。

　　一年来，我们坚持稳中求进，经济实现平稳较快发展。坚定工业集群化、农业产业化、服务业高端化发展方向，加速推进转型升级，实现了增长速度与经济质量、发展效益的同步提升。

　　工业经济稳中有增。全市新增规模以上工业企业20户；规模以上工业增加值实现715亿元，增长5.1%。油气开采和化工产业稳步发展。积极开展地企对接，全面深化战略合作，大力支持吉林油田分公司稳油增气，着力推动37个未动用含油区块加快开发，油气产量当量完成630万吨。油页岩开发试验取得突破性进展。松原石化搬迁改造项目部分装置完成检修。乾源50万立方米LNG项目建成投产，天伦天然气合成焊割气项目开工建设，富德甲醇制烯烃化工基地等项目积极推进。食品和农产品加工产业初具规模。盼盼食品工业园投入试生产，正鑫玉米休闲食品项目开工建设，格力粮食产业园、吉林交投玉米系列食品等项目稳步推进。中粮健康生猪产业化项目二期、正邦200万头生猪养殖加工一体化养殖场、曙光30万吨饲料加工和10万吨玉米收储项目建成投产，齐全5万头种猪繁育和30万吨饲料加工项目一期竣工，特沃斯万头精品肉牛养殖屠宰加工项目开工建设。传统优势及新兴接续产业发展加快。吉正农业机械制造、地都10万吨生物菌肥、黄栀花药业中药加工等项目投入生产，中化长山改扩建项目进入尾声，五环汽车模具及冲压件、金樽家居用品等项目建设快速推进，嘉吉生化扩能改造项目开工建设。聚太生物质发电、三缘光电高科技大功率LED等项目建成投产。科技创新能力明显提升。万吨级秸秆汽爆乙醇产业化技术成果通过中科院鉴定，众诚集团油页岩化学干馏提取技术填补国内原位开采技术空白，大多防腐防垢金属陶瓷内衬油管技术列入国家首批再制造产品目录。清华科技园获国家科技型中小企业科技服务支持，吉东科大新型高效节能超导热锅炉开发等8个项目列入吉林省科技发展计划。

　　农业发展稳中向好。农业增加值实现280亿元，增长5.6%。农业综合生产能力持续增强。哈达山水利枢纽工程主体完工，松原灌区项目通过国家核准，河湖连通和引松入扶工程开工建设。整理土地13.8万亩，新建高标准基本农田17.9万亩。全市首家院士工作站­­成立。打赢抗旱夺丰收保卫战，全市粮食总产量达到713.5万吨。集约高效农业快速发展。积极推广长岭兴源合作社等集约化经营模式，新增农民专业合作社300个，流转土地180万亩。创建粮油高产示范区95万亩，完成膜下滴灌70万亩。发展无公害、绿色和有机农业150万亩，台湾鑫鑫有机农业绿色循环生态产业园一期投入生产。农业产业化进程加快。松粮集团“查干湖”品牌农产品销售收入实现翻番，突破7000万元。乾安弱碱食品开发基地建成，中科微田现代农业产业园项目一期完工。新增棚菜面积0.7万亩，蔬菜自给率提高3个百分点。新建标准化规模养殖场（小区）160个。畜牧业增加值实现80.3亿元，增长7.9%。

　　现代服务业稳中提质。服务业增加值实现650亿元，增长8%。旅游业发展势头强劲。华西希望以“花舞松原”为代表的哈达山示范区综合开发、大连凯伦威尼斯水上乐园项目成功落地。查干湖旅游经济开发区与国家发改委国际合作中心签订战略合作框架协议。查干南湖滨湖风光带建设进入尾声，天河谷温泉旅游度假小镇等项目开工建设。市规划展览馆晋升为国家4A级旅游景区，春捺钵遗址群被确认为全国最大古游牧民季节性渔猎营地。旅游综合收入实现72亿元，增长25%。商贸物流房地产业稳步发展。金钻江南秀、沃尔玛大型连锁超市、馨三百购物中心投入运营，东镇城市广场一期、哈剌和林国际城一期完工。云天化农资物流中心、瑞禾仓储物流中心、正辉煌药业物流等项目建成运营。房地产业总体保持健康发展态势，开发房地产213.2万平方米，销售商品房97.7万平方米。金融业发展步伐加快。城开公司二期11亿元公司债券成功发行，一期10亿元中期票据已上报待批。市中小企业担保公司担保能力放大9倍。交通银行松原支行升格为分行。新增证券公司3户、小额贷款公司14户。金融机构贷款余额增加102.3亿元。

　　一年来，我们深入实施“美丽家园”行动计划，城乡面貌显著改观。结合贯彻落实吉林省西部生态经济区发展战略，突出绿色发展理念和生态文明建设，以“专项行动”为重点，着力打造美丽家园。中心城市建设彰显新风貌。《松原市城市总体规划》修编、城区16个专项规划和53平方公里控详规编制及重要地段城市设计、“智慧城市”中心城区1:500基础地形图测绘及控制测量全面完成。实施重大城市基础设施项目95个，建设规模为历年之最。“一江两岸”建设扎实推进，生态环保型壅水坝和二松城市防洪左岸堤防续建工程全面完工，松花江大桥南岸桥头互通工程竣工通车，天河大桥主桥钢箱梁完成安装。城市道路与地下管网改造统筹实施，郭尔罗斯大路、前程大路、临江西路、青年大街、瓦房街、长宁街等城区街路新建、续建或改建高标准完成；改造排雨排污管网41.2公里，雨污分流率提高20%，有效缓解城市内涝。松原大路、乌兰大街等12条街路和松花江大桥亮化全面完成，城市夜晚更加亮丽多彩。环城绿化带项目启动实施，松江街、沿江路、扶余大路等23条街路和中山公园、五色广场等9处公园广场绿化升级，新增城市绿地2.8万平方米，城市居民享受到更多“绿色福利”。县镇村建设迈出新步伐。县城建设与产业发展加速融合。长山省级特色城镇化示范镇建设扎实推进。以太平川镇马场屯为样板，完成48个省级改善人居环境重点村和230个农村环境提升村建设任务，新建农村公路276公里。立体化交通网建设开启新阶段。松原查干湖机场开工建设。松原客运交通枢纽铁路客运新站房、扶余新客运站投入使用，宁江客运站主体完工，松陶铁路投入运营。省道哈松公路后河拉林至宁江段开工建设。地震灾区建成崭新家园。累计投入资金9亿元，维修房屋15621户，新建房屋7059户，建设学校、卫生院和服务场所等公共基础设施13.7万平方米，房屋损毁户全部及时、安全、温暖、舒心入住新居，灾区恢复重建全面完成。

　　一年来，我们凝聚改革开放正能量，经济社会发展活力不断增强。注重加强顶层部署，统筹推进简政、放权、增活“三位一体”行政体制改革，形成了助推经济社会发展强劲动力。重点领域改革全面推进。新一轮政府机构改革基本完成。在全省率先启动事业单位分类改革、率先实现非行政许可“零审批”。政府行政职权和行政审批事项分别清减25%和44%，清权力度居全省首位。工商登记制度改革全面铺开。不动产登记、“营改增”、农村土地承包经营权确权登记颁证、农村土地收益保证贷款等试点工作顺利开展。第二批县级公立医院综合改革试点成果显著。应收账款质押融资业务全面推广，林权流转抵押贷款试点启动实施。民营经济增势强劲。创建孵化基地4处，发放小微贷款69.5亿元，新增市场主体1.9万户，助推309户个体工商户实现“个转企”。新增中国驰名商标、地理标志证明商标和中国名牌产品6件；新注册商标298件，增幅居全省第一位。民营经济主营业务收入实现2065亿元，增长15.5%。招商引资成果显著。围绕“招大、上新、改旧、扩小”，抽调93名公职人员离岗招商，积极与华西希望、天津宝迪、河北君乐宝、义乌小商品、香港豪德等战略投资者进行高位深层对接，吉林特驱100万头原种猪生猪养殖及食品加工等系列项目和新研农机产业园、大连佐源菊苣加工基地等大项目成功签约。全年引进投资3000万元以上重点项目110个；实际到位资金850亿元，增长5%。实际利用外资3.1亿美元，增长13%。积极争取振兴东北政策支持，油页岩综合利用、松花江干流治理等22个重点项目纳入国省规划。

　　一年来，我们着力保障和改善民生，人民生活持续改善。在总结前两年成功经验基础上，紧紧围绕经济发展惠及民生、设施建设贴近民生、社会事业给力民生，全面完成66件民生实事，实现了推进发展与改善民生的互融联动。民生改善更富成效。城镇登记失业率控制在3.1%，新增城镇就业4.6万人、再就业1.4万人，农村劳动力转移就业68.5万人。城乡居民基本养老保险制度实现并轨，城镇职工和城乡居民养老保险参保人数新增2.3万。12572名“五七家属工”全部纳入养老保险体系。企业退休人员养老金月人均提高151.5元。政府购买居家养老服务试点启动实施，“12349”居家养老信息平台投入使用。医疗保险实现省内异地就医即时结算。城镇居民基本医疗保险报销最高支付限额、新农合住院补助封顶线分别提高10万元和5万元。发放城乡低保资金3.3亿元、各类救助资金4639.6万元。改造棚户区60万平方米，综合整治老旧小区100万平方米，完成“暖房子”工程50万平方米，建设分配廉租房16.9万平方米，改造农村危房14741户。全面兑现“安置遗留户”承诺，720户历史遗留未回迁户全部安置到位。完成18777户回迁楼、油田楼和商品房产权产籍办理。发放住房公积金贷款5亿元。改造背街巷路28条。完成173栋老旧住宅楼供热地沟管网改造，受益居民达8300户。铺设改造天然气管网18公里，新增燃气管道用户2.4万户。撤并老旧小型二次供水站42座，新建大型二次供水站9座，16821户居民喝上“放心水”，彻底解决了主城区居民饮水安全问题。江南、江北污水深度处理项目完成土建工程。新建水冲公厕和垃圾压缩转运站20座。公交车“暖车厢”工程和出租车刷卡乘车试点顺利实施，新建高档公交候车亭30座。社会事业更加繁荣。全市中小学“阳光录取、阳光分班”全面推行。市第二高中综合教学楼主体完工，改造薄弱学校30所，新改扩建公办幼儿园、“儿童之家”17所。建成市级示范性中小学、优秀村级小学40所。临江社区卫生服务中心主体完工，新建标准化村卫生室348所。免费实施白内障复明手术1097例。市精神病院综合楼和市残疾人康复中心、托养中心全面落成。新建社区小广场、村级文化小广场302个。成功举办首届松原辽金文化博览会和首届社区文化艺术节。博舞入选国家级非遗代表性项目名录。完成偏远村屯广播电视“户户通”工程568户。圆满承办查干湖全国公路自行车邀请赛，成功举办奥运冠军走进东北老工业基地暨纪念奥运圣火松原传递6周年健康跑活动。新一代天气雷达完成设备吊装。活断层探测与地震危险性评价全面完成。地震前兆动植物观测站和江北步行街人防工程投入使用。

　　一年来，我们切实加强社会治理，社会保持和谐稳定。全力推进法治松原建设，深入开展“六五”普法，建立国省级民主法治示范村29个、省级法治文化示范基地2个。严格落实信访工作领导包保化解稳控责任，解决历史积案137件。扎实推进平安松原建设，社会治安综合治理成效显著，群众的安全感和满意度持续提升。全面构建“四化融合”、“三位一体”安全生产防控体系，事故起数和死亡人数实现“双下降”。应急平台体系进一步完善，各类突发事件得到及时妥善处置。

　　一年来，我们扎实开展群众路线教育实践活动，政府自身建设不断加强。自觉接受人大法律监督、工作监督和政协民主监督，办理人大代表建议77件、政协提案146件，办复率和满意率均达100%。坚决整治“四风”突出问题，各类会议、文件简报和公务接待数量分别下降18%、20%和30%，腾退超标办公用房3.8万平方米，封存越野车351台，压缩“三公”经费支出5%。强化审计监督和行政监察，严厉整治“吃拿卡要”、“慵懒散奢”等问题，查处违纪违法案件660件。此外，民族宗教、外事侨务、双拥共建、人口计生、食品药品监管、统计、档案、地方志、红十字等工作都取得了新成绩。

　　各位代表！过去一年，面对经济发展新常态和全市人民新期待，我们攻坚克难、奋力拼搏，努力追求有质量的增长和可持续的发展，实现了调结构与增后劲统筹推进、惠民生与优生态同步前行、克时艰与利长远协调并济。过去一年成绩的取得，是省委、省政府和市委正确领导、统揽全局的结果，是市人大和市政协大力支持、有效监督的结果，是全市广大干部群众不畏困难、团结进取的结果，是中省直单位、驻松部队、武警官兵和社会各界积极参与、合力共建的结果！在此，我代表市人民政府，向所有为松原发展作出贡献的同志们、朋友们，表示崇高的敬意和由衷的感谢！

　　各位代表！在看到成绩的同时，我们也清醒地认识到，当前我市经济社会发展还存在一些不容忽视的困难和问题。从发展环境上看，经济发展转型与增长动力转换不协调不同步，转型发展攻坚期恰逢新背景下宏观经济波动期；经济增速持续放缓，融资举债空间收窄；招商引资要素优势减弱，承接项目成本明显加大。从资源利用上看，发展油气化工的资源“不占有”瓶颈仍待突破，丰富的粮食资源尚未转化为高附加值的产业链条，特色旅游业的季节性“真空期”问题亟待解决。从产业结构上看，新的主导产业规模优势尚未形成、产能充分释放尚需时日，战略性新兴产业发展相对较慢，项目谋划建设缺乏战略性思维和链条式设计，契合国家重点投资导向的产业类大项目、好项目偏少，“受制于油”的发展局面短期内仍然难以逆转。同时，保障民生等刚性支出增加与财政增收压力加大的矛盾凸显，政府职能转变还不到位，经济发展环境亟待优化，个别部门服务意识不强、工作落实不力，一些公职人员进取意识不足、存在“为政不为”倾向。对此，我们必须采取有力措施，认真加以解决。

　　二、2015年工作安排

　　2015年是完成“十二五”规划目标收官之年，也是松原转型发展关键之年。我们既面临难得机遇，也面临巨大挑战。一方面，国家稳增长的政策举措将释放更多红利，支持东北地区振兴等一系列利好政策的综合效应发力提速，吉林省西部生态经济区发展战略深入实施。我市作为全国唯一的生物质能源产业集群城市和全省唯一的成长型资源城市，必将在新一轮发展中获得更多倾斜性支持，为我们转型发展、振兴发展提供了难得的历史机遇。另一方面，当前我国经济发展正处于从高速到中高速的增速换挡期、结构调整阵痛期和前期刺激政策消化期“三期叠加”的特殊阶段。今后一个时期，中央对宏观经济政策在保持主基调不变的前提下，将会适时适度作出预调微调，加之国际油价涨跌趋势不明朗，预期今年我市经济发展外部不确定因素增多，下行压力有增无减。

　　基于此，今年政府工作的总体要求是：全面贯彻落实党的十八届四中全会和省委、市委全会精神，积极适应宏观经济新常态，紧紧抓住国家实施新一轮振兴东北和我省建设西部生态经济区战略机遇，以“转型发展、富民强市，建设幸福松原”为工作主题，以稳中求进、稳中求好为发展导向，探索新思路，挖掘新动力，激发新活力，强力推进转型攻坚、增长攻坚、改革攻坚、惠民攻坚，科学施策，开拓进取，努力开创松原经济可持续发展和包容性增长新局面。

　　强力推进“转型攻坚”，准确把握我市资源型经济特点和发展阶段性特征，促进产业结构多元化、增长动力多极化、资源利用链条化、企业发展高端化，加快夯实发展基础，走出“资源依赖怪圈”。强力推进“增长攻坚”，坚持综合提质、稳中求进，全力开展招商引资和项目建设，积极推进油页岩、油田新区块和生物质等资源战略开发，加快培育新的经济增长极，努力使经济增长保持在合理区间。强力推进“改革攻坚”，根据中央、省委全面深化改革顶层设计和市委深化改革实施方案，以创新的思路和有力的举措，进一步破除发展障碍，激发发展活力，汇聚发展能量，构筑经济社会发展新优势。强力推进“惠民攻坚”，以“美丽家园”和民生两个行动计划为载体，突出生态文明建设和人民生活改善，大力提高城乡综合承载力和秀美宜居度，进一步以发展的“红利”增进民生的“福利”，让全市人民的幸福指数不断提升。

　　综合考虑各方面因素，2015年全市经济社会发展主要预期目标是：地区生产总值增长7%左右，地方级财政收入按可比口径增长3%，全社会固定资产投资增长12%，社会消费品零售总额增长10%，城镇和农村常住居民人均可支配收入分别增长3.5%和10%，城镇登记失业率控制在4%以内，万元GDP能耗下降2%。具体做好七个方面重点工作：

　　（一）致力于稳增长，推动经济规模实现新扩张。坚持项目牵动、招商带动、民营联动、金融促动，在力求提质增效的同时，继续保持经济合理增速、适度增量。高效推进项目建设。新建续建3000万元以上项目600个，其中亿元以上项目120个。加快项目建设进度，提高设施类项目建成使用率和产业类项目建成投产率。围绕战略性新兴产业、现代农业、重大基础设施、保障和改善民生、生态环境保护等重点领域，结合编制“十三五”规划，深度谋划一批重大优质项目。着力提高招商引资质量。充分发挥区位、资源等综合优势，瞄准重点地区、重点企业和重要战略投资者，打响新一轮招商引资攻坚战。在招商主体上探索委托招商、战略合作招商等新路径，在招商数量上必保每个县（市区）、开发区引进两个3亿元以上大项目。继续积极引进高科技、高附加值、高回报率、高就业率项目，重点推动河北君乐宝养殖及乳制品加工基地、川酒东北分装储运销基地、北京日化洗涤系列产品、长德商贸物流城等大项目签约落地。全年招商引资到位资金890亿元，增长5%。不断壮大开发区经济。强化开发区招商引资和项目建设主体功能，促进产业加速集群，切实增强创新发展、内涵式发展和引领带动发展能力，加快将经开、石化、查干湖、哈达山和农高新五个开发区分别打造成为全市食品、油气和生物化工、特色旅游、现代农业的“产业航母”。大力发展民营经济。全面落实国家对小微企业支持政策，对信用等级高的企业在政府采购、融资担保、商标培育、技术改造等方面给予重点扶持，加快雅达虹民营企业孵化基地建设，培育500户成长性好的中小企业，扶持100户骨干企业发展壮大。全年实现民营经济主营业务收入2350亿元，增长10%。努力破解资金瓶颈。促进银行业金融机构加大对域内发展支持力度，重点增加对工业项目及小微企业信贷投放比重。争取实现村镇银行县域全覆盖，大力推进宁江惠民村镇银行在新三板挂牌上市，争取宁江、前郭农联社分别改制重组为农商行，新增小额贷款公司5户。积极推进农业直补贷款，扩大农村土地收益保证贷款试点范围。探索通过PPP、BOT等市场化途径实施城市供热、供气、给排水等项目建设，多措并举畅通金融血脉。规范政府融资行为，合理控制债务规模。

　　（二）致力于调结构，推动发展质量获得新提高。坚持以存量巩固发展基础，以增量带动结构优化，着力提升产业丰厚度，加快打造经济“升级版”。

　　推动工业提速增效。新增规模以上工业企业20户；规模以上工业增加值实现765亿元，增长7%。继续做大油气开采产业。进一步深化地企战略合作，大力支持吉林油田和东北油气两家油企稳定生产，全市油气产量当量达到630万吨；积极推动新区块加速开发，确保形成较大增量；促进更多油田配套企业入网经营。加快推进油页岩试验开发，力争进入实质性开采阶段。加快发展油气化工产业。全力推进松原石化搬迁改造，争取150万吨炼油项目投入生产。力争富德甲醇制烯烃化工基地等重大项目开工建设。促进食品和农产品加工业集群发展。推动盼盼食品工业园全面投产，正鑫玉米休闲食品、金地海外5万头荷斯坦奶牛养殖及高端奶制品加工等项目建成运营，开工建设中欧食品产业园、格力粮食产业园、佐源菊苣加工基地、曙光3000万只肉禽屠宰加工等项目，争取嘉吉百亿食品产业园、中粮健康生猪产业化三期和饲料加工等项目启动建设。大力推进新能源和传统产业发展。加快打造生物质能源产业园。启动建设乾安中电风电三期、金地新型建材等项目。推动中化长山改扩建、阳光汽车部件扩建等项目建成投产。开工建设新研农机产业园和史丹利复合肥项目。

　　促进农业提质升级。一产增加值实现295亿元，增长5.4%。加强农业综合生产能力建设。全面发挥哈达山水利枢纽工程作用，开工建设松原灌区，加快推进引松入扶等重大水利工程建设。建设高标准基本农田8万亩，创建粮油高产示范区90万亩，新增膜下滴灌67万亩，新打抗旱井4520眼。正常年景全市粮食总产量达到800万吨。加快现代农业发展步伐。大力支持种粮大户、家庭农场、农村专业合作组织和农业龙头企业发展，积极鼓励农村土地经营权有序流转，新增农民专业合作社300个、市级以上农业产业化龙头企业20户、土地规模经营面积30万亩。加快实施“健康米”工程，打好绿色、有机、精品、高效“四张牌”，深入推进松粮集团与格力集团战略合作，开工建设中科微田现代农业产业园二期项目，台湾鑫鑫有机农业绿色循环生态产业园增加水稻种植面积1万亩，优质马铃薯、葵花、花生等经济作物种植面积达到230万亩。新增棚菜面积0.5万亩，进一步提高反季节蔬菜自给率。积极推进牧业强市工程。结合落实省政府现代畜牧业“四年提升计划”，大力实施“草变肉”、“粮变肉”工程。齐全5万头种猪繁育项目二期竣工，开工建设吉林特驱100万头原种猪生猪养殖项目。新建标准化规模养殖场（小区）160个。实现畜牧业增加值85亿元，增长6%。

　　促进服务业提档扩量。继续保持服务业发展强劲势头，实现增加值705亿元，增长8.5%。突出发展特色旅游业。完成全市旅游总体规划修编和天河谷温泉旅游区、鳇鱼圈满族风情园、三江口湿地公园等景区规划编制。加快推进查干湖、哈达山、春捺钵遗址群、大布苏泥林、大金碑等景区景点综合开发，开工建设威尼斯水上乐园，启动查干湖生态产业园项目一期建设，完成华西希望哈达山示范区综合开发项目规划设计。推进旅游与生态、民俗、文化深度融合，大力发展避暑度假、农业观光、乡情体验等旅游项目，着眼于融入东北地区大旅游格局，构建“四季有景、各具特色、内涵丰富、故事精彩”的开放式精品旅游线路。全年实现旅游综合收入90亿元，增长25%。着力发展现代商贸物流业。镜湖半岛商贸综合体、两江农产品加工及仓储物流等项目投入运营，开工建设东镇城市广场二期、长岭物流开发区等项目。依托区位和交通优势，规划建设粮食等区域性大型集散中心。稳健发展房地产业。合理调控开发节奏，开发房地产100万平方米。全面落实住房消费各项政策，着力激活商品房市场。同时，积极发展总部经济、电子商务、连锁经营等新型业态，推动服务业向高端升级。

　　（三）致力于促改革，推动发展活力得到新增强。紧紧围绕重要领域和关键环节，强力推进各项改革取得实质性进展和明显成效。深化行政体制改革。继续取消和下放行政审批事项，加快实施行政审批清单制度。深入推进事业单位分类改革，开展建立法人治理结构试点。科学整合不动产登记部门职能，最大限度减少职责交叉分散。加快行政执法体制改革。完善食品药品监管体系。完成党政机关公务用车制度改革。推进经济体制改革。分类推进国有企业改革，大力发展混合所有制经济。深化财政管理体制改革，完善预决算公开和国库集中支付等制度。继续推进工商注册制度便利化。扩大农村土地承包经营权确权登记颁证试点范围。加快建立农村土地和林地经营权流转市场体系。争取国家和省在我市开展农村宅基地流转试点。深化粮食流通体制改革。开展供销社综合改革试点。加快社会领域改革。大力推进户籍制度改革。全面推进素质教育综合改革和职业院校教育教学深化改革。争取我市纳入国家养老服务业试点。着力推进全市县级公立医院综合改革全覆盖，巩固完善基本药物制度和基层医疗卫生机构运行新机制，积极推动社会办医，启动城乡居民基本医疗保险制度整合工作。

　　（四）致力于强功能，推动城乡建设彰显新风貌。以打造哈长城市群重要节点城市为目标，扎实推进“以人为核心”的新型城镇化，建设更加宜居的美丽家园。全面强化规划引领。着眼于构建“一带一心、两翼多点”的城镇发展格局，统筹推进各县（市、区）新型城镇化规划编制，完成城市综合交通、城市地下空间利用等专项规划编制，启动实施棚户区改造控详规及修建性方案设计，进一步扩大中心城区控详规覆盖面，不断优化城乡空间布局。扎实推进城乡建设。加快“一江两岸”建设步伐，推进松花江流域两岸综合治理，启动江南城区圣湖补水工程，实施滨江大道延伸工程，开工建设江心岛公园和纳仁汗公园二期。实施松原大路、发展大路、建华路、环保大街、溪浪河大街、伯都讷北街等街路延伸、改造或新建，进一步完善城区路网。开工建设环城绿化带。加快沿江西路、青年大街、学府街等主街路绿化美化亮化升级。江南、江北污水深度处理及污泥干化项目投入运行。实施文化路泵站、哈达大街雨水管线等设施改造。稳步推进扶余、长岭、乾安县城和长山、三井子等重点特色镇基础设施建设，深入实施新农村建设“六大工程”，新建农村公路150公里。着力提高城市管理现代化水平。加快“智慧城市”建设步伐，完成“数字松原”地理空间框架工程。制定出台制度化、科学化、精细化城市管理办法，大力开展环境卫生和道路交通综合整治，保持城市环境整洁舒适常态化。

　　（五）致力于惠民生，推动人民生活取得新改善。像抓经济建设一样抓民生保障，像落实发展指标一样落实民生任务，紧紧围绕生存性、安全性和发展性三大民生需求，以全力办好十个方面75件民生实事为重点，推动民生工作从点向面、从个案向普惠、从物质向精神层面拓展延伸，努力求取改善民生最大公约数。推进富民增收行动。新增城镇就业4.5万人、再就业1.4万人，保持城镇“零就业家庭”动态为零。农村劳动力转移就业50万人。发放小额担保贷款5000万元。推进保障救助行动。新增城镇职工基本养老保险参保人员1万人、城乡居民养老保险参保人员2万人。进一步扩大医疗、工伤、失业和生育保险覆盖面。继续提高城乡低保标准。大力实施慈善大病救助和慈善助学工程。启动“救急难”救助工作。扩大政府购买居家养老服务范围，新建4所城市养老社会福利中心，加快农村居家养老服务大院建设。推进百姓安居行动。改造棚户区55万平方米，建设安置住房7744套。完成松苑雅居廉租房建设和分配。综合整治老旧小区57万平方米，对67栋单体楼、弃管楼和老旧小区实施综合管理。完成300万平方米无籍房产权登记。改造农村危房10635户。推进市政公用行动。新建大型二次供水站2座。加快江北净水厂哈达山取水工程建设。解决10.8万农村居民饮水安全问题。改造120栋老旧住宅楼供热地沟管网，改善7000户居民取暖条件。铺设天然气管网10公里，新增天然气用户1万户。加快实施老旧供电设施改造工程。改造背街巷路14条。完成2处公交停车场建设，更新市区1路、2路公交车。布局建设江南、江北零散用工劳务市场。新建环卫工人爱心屋50个。推进教育提升行动。大力发展职业教育。油田第十一中学、前炼小学综合教学楼投入使用，新建扩建农村幼儿园11所，建设“儿童之家”22所，实施部分农村学校教室建设和校舍维修工程。推进卫生医疗行动。加快推进市中心医院异地新建、市儿童医院项目建设，启动市妇幼保健院和市生殖保健院新建，改扩建8所乡镇卫生院，新建371所标准化村卫生室。免费为全市计划怀孕夫妇提供孕前优生健康检查。做好油田医保属地化管理承接工作。推进文体繁荣行动。市体育馆投入使用，改建江北体育场，新建全民健身场地设施26处、农村文化小广场260个。免费为农村居民放映电影13400场。推进环境改善行动。全面完成主要污染物减排任务，加强对大气、水及石油开发污染监管，推进烟粉尘达标治理和机动车尾气污染防控工程，垃圾焚烧发电项目投入使用。认真抓好林地清退和草原荒漠化治理，扎实推进查干湖生态环保试点项目建设。完成300个村“四清”、“四改”任务，整治290个村人居环境，绿化村屯28个。推进平安创建行动。中心城区新增10个夜间巡逻点位、100个治安高清视频监控点。完成市区交通路口渠化32处，重新施划商圈和重点路段停车泊位1500个。开工建设市食品检验检测中心。推进基础设施行动。扎实推进松原查干湖机场、松原客运交通枢纽与站前综合改造、天河大桥、河湖连通项目建设，宁江客运站投入运营，开工建设建华路下穿桥梁、成吉思汗大街下穿铁路桥、五环大街公铁立交桥、长白铁路扩能改造、长春至西巴彦花铁路松原段、五右高速松原至扶余段等项目，省道哈松公路后河拉林至宁江段建成通车。

　　（六）致力于维稳定，推动社会治理达到新水平。扎实推进依法治市，全面加强社会治理，确保社会和谐稳定。突出抓好安全生产。认真落实“三个责任”，加强标准化建设和网格化管理，严格执行重大安全隐患挂牌督办制度，坚决遏制重特大事故发生。加强社会综合治理。加快法治松原建设，深入开展“六五”普法、“法律六进”和“四级联治联创”活动。妥善处理征地、拆迁等涉及群众切身利益问题，依法依规维护群众合法权益。完善涉法涉诉信访依法终结制度，初信初访办结率达到95%以上。有效维护公共安全。夯实立体化治安防控体系，不断加大对各类犯罪活动打击力度。全面加强流动人口和特殊人群管理。严格强化网络监管。加大食品药品安全监管力度。加强应急管理工作，有效预防和妥善处置各类突发事件。

　　（七）致力于塑形象，推动政府建设实现新转变。结合建立群众路线教育实践活动长效机制和“德润松原”建设，以锲而不舍、驰而不息的精神，扎实推进政府自身建设，努力打造人民满意的“五型”政府。坚持依法行政，建设法治型政府。积极主动接受市人大及其常委会法律监督、工作监督和市政协民主监督。进一步完善面复制度，继续认真办好人大代表议案、建议和政协提案。依法履行政府职能，完善政府涉法事项风险防范机制，坚决把公众参与、专家论证、风险评估、合法性审查和集体讨论决定作为重大行政决策法定程序，确保一切行政行为于法有据、民主科学。建立行政裁量基准制度，压缩行政处罚自由裁量空间，让违法行政在法治的天空下失去生存土壤。坚持笃实为政，建设诚信型政府。推进政府信息公开，完善新闻发布制度，切实提高政府工作透明度。倍加珍惜政府诚信资本，向投资者和老百姓作出承诺做到言必行、行必果，以政府公信带动社会诚信，以“信用松原”集聚人气、汇集商气。坚持为民执政，建设服务型政府。突出服务角色和便民利民，大力推进简政放权，加快政府职能转变。更加认真倾听群众呼声，更加努力解决群众合理诉求。严厉整治“吃拿卡要”等行为，切实开展好政行风评议，大力改善经济发展环境。坚持高效理政，建设效能型政府。深入开展机关作风建设，继续精简会议、压缩文件、取消不必要的检查评比和总结表彰等活动，把主要精力用在办实事、抓落实上。严整“慵懒散浮”，重治“为政不为”，强化目标管理、过程督查和绩效考核，不断提高政府工作执行力和效能。坚持从严治政，建设廉洁型政府。严格执行各项规定，从严控制“三公”经费支出，坚决遏制奢靡享乐之风。认真落实党风廉政建设责任制，切实加强对重点领域、关键岗位的行政监察和审计监督，坚决查处各类违纪违法案件，坚持不懈地营造风清气正的政务环境。

　　各位代表!

　　当前的松原，正处于振兴发展的关键时期！展望新的一年，爬坡过坎、竞相发展，形势逼人奋进；克难制胜、再创佳绩，我们信心满怀！让我们在市委的坚强领导下，抢抓机遇，迎难而上，埋头苦干，开拓创新，为实现“转型发展、富民强市，建设幸福松原”的宏伟目标而努力奋斗！
